# Quantitative Magnetization Transfer Imaging of the Breast at 3.0 T: Reproducibility in Healthy Volunteers

**DOI:** 10.18383/j.tom.2016.00142

**Published:** 2016-12

**Authors:** Lori R. Arlinghaus, Richard D. Dortch, Jennifer G. Whisenant, Hakmook Kang, Richard G. Abramson, Thomas E. Yankeelov

**Affiliations:** 1 Vanderbilt University Institute of Imaging Science, Vanderbilt University Medical Center, Nashville, Tennessee;; 2 Department of Radiology and Radiological Sciences, Vanderbilt University Medical Center, Nashville, Tennessee;; 3 Department of Biomedical Engineering, Vanderbilt University, Nashville, Tennessee;; 4 Vanderbilt-Ingram Cancer Center, Vanderbilt University Medical Center, Nashville, Tennessee;; 5 Department of Biostatistics, Vanderbilt University Medical Center, Nashville, Tennessee;; 6 Center for Quantitative Sciences, Vanderbilt University Medical Center, Nashville, Tennessee;; 7 Department of Biomedical Engineering, The University of Texas at Austin, Austin, Texas;; 8 Department of Internal Medicine, The University of Texas at Austin, Austin, Texas;; 9 Institute for Computational and Engineering Sciences, The University of Texas at Austin, Austin, Texas; and; 10 Livestrong Cancer Institutes, The University of Texas at Austin, Austin, Texas

**Keywords:** quantitative MR, breast cancer, pool size ratio, test–retest

## Abstract

Quantitative magnetization transfer magnetic resonance imaging provides a means for indirectly detecting changes in the macromolecular content of tissue noninvasively. A potential application is the diagnosis and assessment of treatment response in breast cancer; however, before quantitative magnetization transfer imaging can be reliably used in such settings, the technique's reproducibility in healthy breast tissue must be established. Thus, this study aims to establish the reproducibility of the measurement of the macromolecular-to-free water proton pool size ratio (PSR) in healthy fibroglandular (FG) breast tissue. Thirteen women with no history of breast disease were scanned twice within a single scanning session, with repositioning between scans. Eleven women had appreciable FG tissue for test–retest measurements. Mean PSR values for the FG tissue ranged from 9.5% to 16.7%. The absolute value of the difference between 2 mean PSR measurements for each volunteer ranged from 0.1% to 2.1%. The 95% confidence interval for the mean difference was ±0.75%, and the repeatability value was 2.39%. These results indicate that the expected measurement variability would be ±0.75% for a cohort of a similar size and would be ±2.39% for an individual, suggesting that future studies of change in PSR in patients with breast cancer are feasible.

## Introduction

Because of the complicated nature of tumors and their microenvironment (1), a variety of quantitative imaging techniques, each probing unique aspects of the tissue, are required to provide a more complete picture of the changes observed in both the diagnostic and prognostic settings. In addition, as the number of targeted cancer therapies increases (2), it is important to have corresponding imaging techniques that are sensitive to the specific changes induced by such therapies. Two major aspects of tumor physiology that have been studied clinically with magnetic resonance imaging (MRI) are tumor vasculature (3) and cellularity (4). Dynamic contrast-enhanced MRI (DCE-MRI) provides information related to tumor vasculature, making it useful for assessing antiangiogenic therapies, whereas diffusion-weighted MRI is sensitive to tumor cellularity, making it useful for assessing cytotoxic therapies. Recently, we reported that combining DCE-MRI and diffusion-weighted MRI measures increases the ability to predict breast cancer response to neoadjuvant therapy at a very early time point (5). However, although the measurements were relatively sensitive (92%), they were only modestly specific (78%), warranting investigation of other imaging parameters reflecting additional aspects of the tumor environment to more accurately predict treatment response.

Another aspect of the tumor environment that has recently gained increasing attention is the extracellular matrix (ECM) (6, 7). Once thought to be a passive medium, the ECM is now known to be involved in both tumor development and progression (1, 8, 9). The ECM comprises several glycoproteins, including collagen, laminin, proteoglycans, and fibronectin. Although the low concentration and fast relaxation of the protons of these macromolecules make them difficult to image directly with conventional MRI methods (because of their short *T*_2_), their effects on free water can be explored via magnetization transfer MRI (MT-MRI) (10).

MT-MRI is sensitive to changes in the macromolecular content of tissue and takes advantage of the fact that macromolecular protons communicate their spin information to protons in the surrounding free water through dipole–dipole interactions and/or chemical exchange. The magnetization transfer (MT) effect is conventionally quantified by the MT ratio (MTR): MTR = 1 − *M*_sat_/*M*_0_, where *M*_sat_ is the MT-weighted image and *M*_0_ is the reference image with no MT-weighting.

Initial applications of MT-MRI in breast cancer showed a significant reduction of MTR in malignancies compared with benign lesions (11, 12). Bonini et al. (11) hypothesized that increased proteolytic activity may cause a reduction of macromolecules in the malignant tumor environment, thus resulting in reduced MTR. Furthermore, it has been shown that the concentration of collagen type I, the primary structural component of breast ECM, is reduced in the hypoxic tumor environment (13). ECM characteristics are known to change during the tumor life cycle and in response to treatment (14–16); thus, it is a reasonable hypothesis that the MTR may change during the course of therapy and potentially be useful for predicting treatment response. However, MTR measurements are sensitive to the experimental design, making longitudinal comparison difficult. More specifically, both the method of saturation of the macromolecular proton pool (power, offset frequency, and duty cycle) and the image acquisition parameters (repetition time [TR], echo time [TE], and flip angle) affect the observed MTR values. In addition, MT-MRI is truly only a semiquantitative imaging technique, as MTR measurements are affected by both the relaxation and exchange rates of the free water and macromolecular proton pools. Therefore, a quantitative MT (qMT) approach is necessary to separate the contributions from MT and relaxation effects (17, 18) and, ideally, provide more specific information about tissue composition than the MTR.

Although reports of qMT imaging of tumors (19–23) and changes to the extracelluar environment due to disease (23, 24) are limited, these efforts suggest that the macromolecular-to-free water proton pool size ratio (PSR) differs between healthy tissue and tumors or fibrotic tissue. We, therefore, hypothesize that the PSR of breast tumors, as measured with qMT, will be altered compared with the surrounding fibroglandular (FG) tissue and will change in response to successful therapy, potentially improving prediction of treatment response in an ongoing clinical trial (5). However, to assess the sensitivity of qMT imaging in treatment prognosis, an expectation of the variation in the PSR of healthy breast tissue is necessary. Thus, in this contribution, we sought to determine the reproducibility of PSR measurements of the breast in healthy controls.

## Methodology

### Subjects

Thirteen women with no history of breast disease were scanned twice within a single scanning session with a 5- to 10-minute break between scans, allowing the volunteers to stretch. Two volunteers did not have any appreciable FG tissue, leaving 11 data sets for analysis (ages: 25–54 years; mean: 33 years). Subjects were consented as part of a study approved by the local Institutional Review Board. Demographic data were collected and managed using REDCap (Research Electronic Data Capture) electronic data capture tools hosted at the Vanderbilt University (25).

### Magnetic Resonance Imaging

Data were acquired with a 3.0 T Achieva MR scanner equipped with a 2-channel multitransmit body coil and a MammoTrak table that includes a dedicated 16-channel receive double-breast coil (Philips Healthcare, Best, The Netherlands). The MammoTrak table (Philips Healthcare, Best, The Netherlands) automatically places the breast coil at the magnet isocenter. Image-based radiofrequency (RF) and *B*_0_ shimming were performed using the SmartBreast software package (Philips Healthcare, Best, The Netherlands).

For qMT imaging, an MT-prepared (20-milliseconds sinc-Gauss pulse), segmented echo planar imaging sequence (3-dimensional gradient echo with 5 lines/shot) with a water-selective excitation pulse (1-3-3-1 binomial, 6°), TR/TE = 48/6.6 milliseconds, sensitivity encoding factor = 1.5, flow-compensation, and respiratory gating was used. A sagittal volume was acquired with a field of view (FOV) = 256 × 256 × 50 mm^3^, acquisition matrix = 128 × 126 × 10 sections, and a reconstructed voxel size = 1.33 × 1.33 × 5 mm^3^. This FOV was centered on the left breast, with an attempt to approximately match the stack placement between scan sessions. [Slice orientation, FOV, and voxel size were chosen to coordinate with those being acquired in an ongoing longitudinal, multiparametric study of response prediction in breast cancer (5).] Data were collected at 4 MT-offset frequencies (1, 2, 4 and 8 kHz) using 2 MT pulse angles (500° and 800°), plus 1 acquisition for normalization (offset frequency = 100 kHz and pulse angle = 800°), resulting in a total of 9 image volumes in a (minimum) scan time of 1 minute 38 seconds. (The actual scan time varied depending on the volunteer's respiration rate and was typically ∼3 minutes.)

The qMT model requires independent *T*_1_, RF transmit (*B*_1_^+^), and main magnetic field (Δ*B*_0_) estimates. *T*_1_ was estimated using the multiple flip angle method with 10 flip angles (2:2:20°), TR/TE = 7.9/4.6 milliseconds, and a matrix size of 192 × 192 with a 192 × 192 reconstruction. *B*_1_^+^ was measured using a Bloch–Siegert method (26), with TR/TE = 491/5.4 milliseconds and a matrix size of 104 × 102 with a 192 × 192 reconstruction. Δ*B*_0_ was measured using a dual-gradient echo method with fat and water protons in phase with TR/TE = 12/4.6 milliseconds and a matrix size of 84 × 85 with a 192 × 192 reconstruction. Each measurement was acquired with the same FOV as the qMT data (256 × 256 × 50 mm^3^) and 10 sections. Scan times for the multiple flip angle, *B*_1_^+^, and *B*_0_ maps were 1 minute 7 seconds, 1 minute 44 seconds, and 9 seconds, respectively.

### Data Analysis

All data were nonrigidly coregistered to the normalization qMT image volume (Advanced Normalization Tools, Philadelphia, Pennsylvania). FG tissue volumes of interest (VOIs) were extracted from each reference qMT image volume in a semiautomated 3-step process (27). First, a threshold-binning procedure eliminated the background noise and voxels affected by partial-volume averaging. Next, the skin and chest wall were manually excluded from the VOI. Finally, the first and last sections were excluded to remove potential interpolation artifacts due to the image registration process.

The registered and masked data were fit to a 2-pool model to estimate PSR and the *T*_2_ of the macromolecular protons (*T*_2_^M^) (28, 29). During fitting, the *T*_1_/*T*_2_ of water protons and the MT rate were fixed to the following published values in skeletal muscle: 40 and 48 Hz, respectively (29). This model reduction was necessary because the number of offsets/angles acquired was limited by the longer scan times associated with respiratory gating. Previous work using similar qMT model reduction strategies has shown that estimated PSR values are relatively insensitive to errors in the assumed values for the *T*_1_/*T*_2_ of water protons and the MT rate (22, 29, 30).

Mean PSR (mPSR) and mean *T*_2_^M^ (m*T*_2_^M^) values for FG tissue were calculated for each scanning session for each patient by averaging the PSR values within the corresponding VOI. PSR values greater than 30% were considered nonphysiological, and voxels exceeding that threshold were excluded from the analysis. These voxels were located primarily at tissue boundaries, where partial-volume effects may lead to erroneous fit values.

Statistical analyses were performed using the statistical toolbox in MATLAB 2007b (The MathWorks, Natick, Massachusetts). Previously published methods (31) based upon the work outlined by Galbraith et al. (32) were used to assess the reproducibility of mPSR measurements in the FG tissue of healthy controls. For each volunteer, the difference, *d*, between the measurement of mPSR from the first scanning session (mPSR_1_) and mPSR from the second scanning session (mPSR_2_) was calculated: *d* = mPSR_2_ − mPSR_1_. A Kendall's tau test was performed to ensure that the measurement error was not correlated with the mean, and the following statistical measurements of reproducibility were then computed: the 95% confidence interval (CI), the root-mean-squared deviation, the within-subject standard deviation, and the repeatability value (*r*).

An additional Kendall's tau test was performed to test for correlation between the average of the mPSR values and age, as it is known that breast tissue composition changes with age (33). Changes in *T*_2_^M^ with treatment response are not expected, as it has been shown that *T*_2_^M^ values are similar in both healthy and diseased tissues (29, 34); therefore, repeatability measures were not performed for the m*T*_2_^M^ values.

## Results

**Figure 1.**
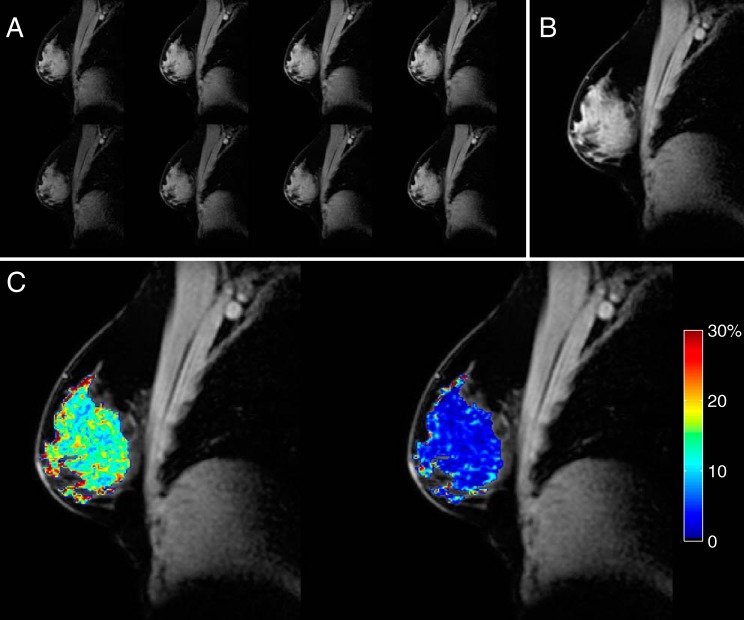
Quantitative magnetization transfer magnetic resonance imaging (qMT-MRI) data are shown for a typical subject (age = 26 years): the 8 magnetization transfer (MT)-weighted images, with pulse angles of 500° (top row) and 800° (bottom row) and offsets of 1, 2, 4, and 8 kHz (left to right) (A); the normalization image (offset frequency = 100 kHz and pulse angle = 800°) (B); and the corresponding pool size ratio (PSR) map (left) and map of the standard deviation (SD) of the PSR values (right) (C) calculated during the fitting process. The mPSR ± SD for this subject and scanning session was 14.5% ± 5.9%.

**Figure 2.**
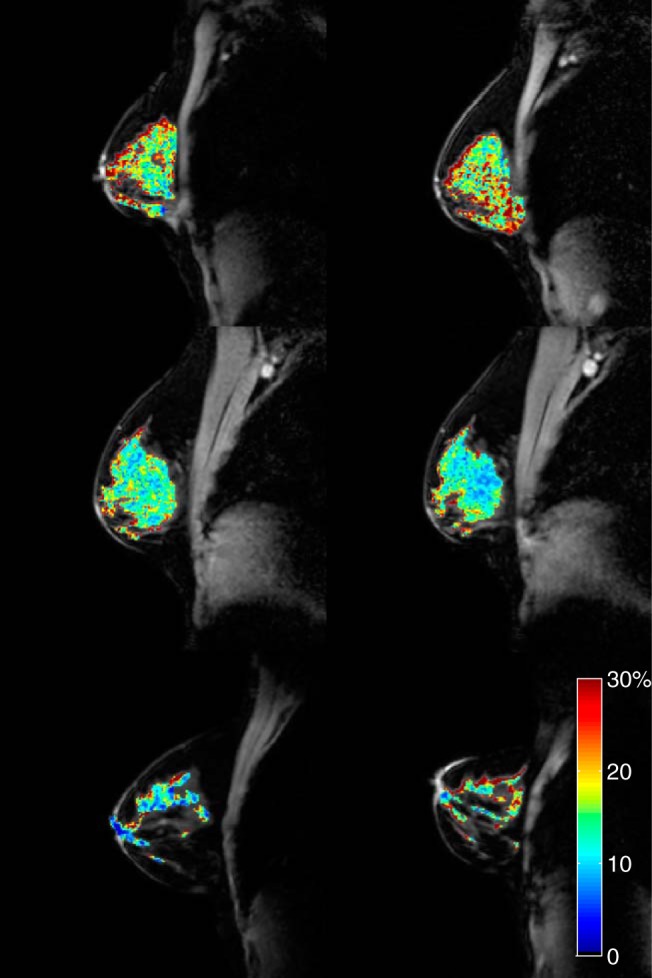
Reproducibility in healthy controls. From top to bottom, each row displays examples of the best (|*d*| = 0.1%), average (|*d*| = 0.9%), and the worst (|*d*| = 2.1%) cases, based on the absolute value of the difference (|*d*|) between the mean fibroglandular (FG) PSR values from scan 1 (left column) to scan 2 (right column). The PSR maps are displayed as overlays on the corresponding normalization images.

**Figure 3.**
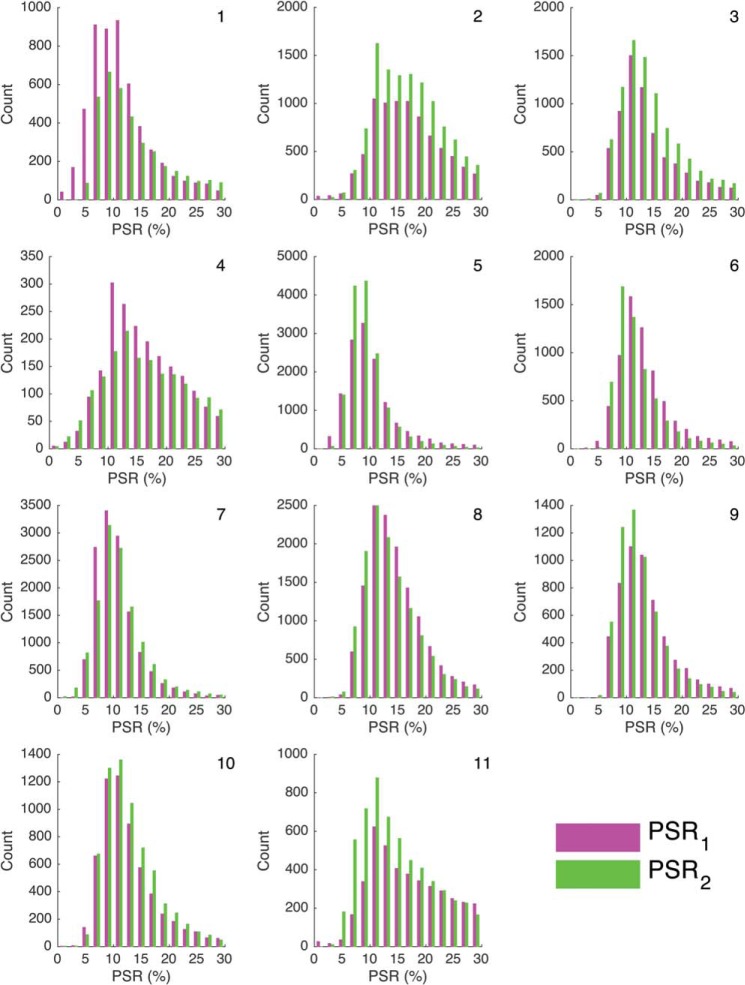
Histograms of PSR values for all FG voxels are plotted by scan session (magenta: first scan, green: second scan) for each of the 11 subjects.

Representative images from a single volunteer are shown in Figure 1. The saturated images (Figure 1A) and the reference image (Figure 1B) show robust fat suppression. The corresponding PSR map is shown as an overlay on the reference image (Figure 1C), showing a distribution of PSR values within the FG tissue. A map of the standard deviation of the fitted PSR values is also shown (Figure 1C) to provide an example of the estimated error in the nonlinear fit. Values of m*T*_2_^M^ and mPSR for each scanning session and the difference, *d*, between the 2 mPSR values are listed in Table 1. The value of m*T*_2_^M^ ranged from 2.7 to 3.9 μs and 2.6 to 10.3 μs for scans 1 and 2, respectively. The value of mPSR_1_ ranged from 10.4% to 16.7%, the value of mPSR_2_ ranged from 9.5% to 16.7%, and the absolute value of *d* ranged from 0.1% to 2.1%. PRS_1_ and PSR_2_ maps for the best (|d| = 0.1%), average (|*d*| = 0.9%), and the worst (|*d*| = 2.1%) cases are shown in Figure 2. Histograms of PSR values for the individual voxels in the VOIs are plotted by the scan session in Figure 3. There was no significant correlation between the average of the 2 mPSR values and age (Kendall's tau, *P* = .273), as can be seen upon visual inspection of Figure 3.

**Table 1.** Age, *T*_2_^M^, and PSR Data

Subject No.		m*T*_2_^M^(μs)		mPSR (%)		
	Age	Scan 1	Scan 2	Scan 1	Scan 2	*d*
1	42	3.6	3.4	11.3	13.4	2.1
2	51	3.1	3.2	16.7	16.7	0.1
3	29	3.0	2.9	13.8	14.2	0.5
4	54	3.9	10.3	16.0	16.2	0.2
5	36	2.8	2.9	10.4	9.5	−0.9
6	27	2.9	3.1	13.3	11.9	−1.4
7	25	3.1	2.6	10.6	11.1	0.5
8	26	3.0	3.1	14.5	13.6	−0.9
9	36	2.9	3.3	13.6	12.5	−1.1
10	27	2.7	2.9	12.6	12.9	0.4
11	33	3.9	5.0	16.7	14.8	−1.9

**Figure 4.**
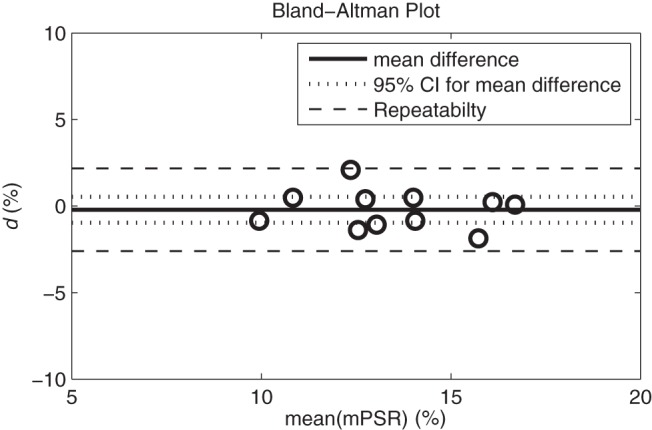
Difference (*d*) between mean PSR (mPSR) values in the FG tissue from 2 scanning sessions plotted against the average of the 2 FG mPSR values for each volunteer. The overall mean difference between scans (solid line) for all 11 subjects was not significantly different from 0 (*P* = .543). The 95% confidence interval (CI) for the group (dotted line) was 0.75, and it represents the level of change that would be significant for a group of 11 subjects. The repeatability (dashed line) was 2.39, and it represents the level of change that would be significant in an individual.

There was no significant dependence of *d* on the mean value of the 2 measurements (Kendall's tau, *P* = .165). The values of *d* are plotted against the mean of mPSR_1_ and mPSR_2_ for each subject in the Bland–Altman plot as shown in Figure 4. The mean difference for all volunteers was −0.2%, which was not significantly different from 0 (*t*-test, *P* = .543). The 95% CI for the mean difference was ±0.75%, root-mean-squared deviation was 1.09%, within-subject standard deviation was 0.77%, and repeatability value (*r*) was 2.39%.

## Discussion

To the best of our knowledge, this is the first report on PSR measurements of FG tissue in the breast in vivo. This study shows that the PSR of healthy FG tissue can be reliably measured using the sequence protocols and analysis pipelines described herein. Of particular note, robust fat suppression was achieved via a water-selective excitation pulse, minimizing the potential influence of fat on PSR values in the breast tissue; the semiautomated VOI selection algorithm was reliable across scan sessions; and the sequence can be performed in a clinically feasible scan time. The 95% CI for the mean difference was ±0.75%, meaning that a change in mPSR greater than ±0.75% would exceed the expected measurement variability for a group of 11 patients. The repeatability value, *r*, was 2.39%, meaning that a change in PSR greater than ±2.39% would exceed the expected measurement variability for an individual. The relatively small interscan variability observed in this small cohort supports continued investigation into the use of PSR measurements in future longitudinal studies of breast cancer progression and/or treatment response.

Currently, there is a paucity of data on MT imaging of the breast in the literature. Santyr et al. (35) performed in vitro studies to assess the MT rates between solid- and liquid-like pools in different agar gels and excised FG specimens. These data, in combination with a theoretical MT model, were used to optimize an MT sequence appropriate for in vivo imaging. They showed that their technique could result in 40%–50% decrease in FG tissue signal, thereby making lesion visualization easier. Based on these data, in vivo MT imaging of the breast was first implemented to simply improve visualization of tissue enhancement after the injection of a standard gadolinium-based contrast agent (36, 37). Pierce et al. (36) showed a 30% reduction in signal with MT-weighting in 2 volunteers and noted improved tumor enhancement in patients with a variety of breast pathologies. Schreiber et al. (37) noted improved visualization of tissue enhancement in MT-weighted images after the injection of a standard gadolinium-based contrast agent. Callicott et al. (38) assessed *T*_1_ and MT properties on breast tissue samples ex vivo. Although these MT methods were able to provide greater discrimination between lesions and the surrounding normal tissue, they were unable to reliably separate benign from malignant neoplasms. In contrast to these more qualitative approaches, Bonini et al. (11) and Heller et al. (12) were able to separate benign and malignant breast lesions by showing a significant reduction of MTR values in malignant cases. Bonini et al. (11) hypothesized that these findings were because of increased proteolytic activity and/or decreased activity of enzyme inhibitors in cancer, both of which may act to reduce the available macromolecular pool. However, those studies (like ours) were not designed to elucidate the underlying biochemical processes resulting in measured changes.

Decreased PSR values have been reported in gliomas in animal models (19, 20, 39), perhaps because of the increased water content within the tumor. Tozer et al. (40) reported reduced macromolecular pool fraction in human gliomas compared with normal-appearing white matter. Thus, given these previously published reports and the data presented here, future qMT-MRI studies designed to study the effects of various biophysical processes in breast cancer on PSR measurements are warranted.

The application of qMT-MRI to cancer imaging, in general, and to the current study is not without its limitations. The entire macromolecular pool affects measured PSR values, making it impossible to correlate changes observed in PSR with changes in the concentration of specific glycoproteins, and changes in PSR values are also affected by changes in water content (eg, inflammation or edema). In addition, the relationship between FG composition and PSR measurements is currently not fully understood. Although FG composition is known to change with age and menstrual status, age does not appear to affect the repeatability measures in this study, as seen in Figure 3 and Table 1; furthermore, Clendenen et al. (41) recently reported that FG MTR values do not vary significantly across the different phases of the menstrual cycle. Variation due to the menstrual cycle was controlled for in the present work by scanning each volunteer within the same scan session, and this may not be an issue in the clinical setting as patients often experience chemotherapy-induced amenorrhea (42).

Another potential limitation of qMT-MRI is that accurate estimation of model parameters requires the acquisition of several image volumes at multiple offset frequencies and/or powers, potentially making the scan prohibitively long. Two ways to overcome this challenge are to reduce the number of image acquisitions by designing optimal sampling strategies (43) and to fix certain model parameters in the fitting procedure (20). As an initial attempt, we selected a combination of 4 offset frequencies and 2 powers for a total of 8 MT-weighted image volumes to reduce the acquisition time and allow for respiratory gating. We also chose to fix the *T*_1_/*T*_2_ of water protons and the MT rate to values previously reported for muscle (29); however, future work may include modeling to estimate the bias introduced by these parameter assumptions.

The pulsed saturation technique used here requires additional image acquisitions (*T*_1_, *B*_1_^+^, and Δ*B*_0_), potentially increasing the total scan time for an imaging session (18, 22, 44); however, a pulsed saturation technique was chosen for this study for 2 reasons. First, it is the most practical for clinical application because it can be applied within the hardware constraints of clinical systems and within patient safety limits for RF power deposition. Second, the protocol for the clinical trial (5), in which we propose to add qMT imaging, currently includes *T*_1_ and *B*_1_^+^acquisitions for DCE-MRI analysis, meaning the only additional scan time is for the Δ*B*_0_ mapping, which takes less than 10 seconds.

The dependence of the pulsed saturation technique on 3 additional image acquisitions also raises the concern that each additional acquisition might introduce bias into PSR estimation. However, the methods applied in this study attempt to mitigate those effects. First, the lowest offset frequency applied in this study was 1 kHz; therefore, errors in *B*_0_ on the order of 50 Hz (45) would result in negligible errors in PSR (simulations not shown). Second, PSR measurements are effectively insensitive to *B*_1_^+^ error when the *B*_1_^+^ map is used for both the correction of the *T*_1_ mapping and the qMT analysis (46), as was done in this effort. Finally, PSR bias scales linearly with errors in 1/*T*_1_, and the *T*_1_ mapping method used in this work, which included *B*_1_ correction, has been shown to produce reliable results (47).

In conclusion, the results of this study demonstrate the feasibility of performing qMT-MRI of the breast in healthy controls using a pulsed saturation technique. PSR measurements of the FG tissue estimated with a 2-pool model were reproducible over 2 scan sessions. Future work includes applying the technique in an ongoing longitudinal, multiparametric study of treatment assessment in breast cancer (5).
